# Characterization of Transglutaminase 2 activity inhibitors in monocytes *in vitro* and their effect in a mouse model for multiple sclerosis

**DOI:** 10.1371/journal.pone.0196433

**Published:** 2018-04-24

**Authors:** Navina L. Chrobok, John G. J. M. Bol, Cornelis A. Jongenelen, John J. P. Brevé, Said El Alaoui, Micha M. M. Wilhelmus, Benjamin Drukarch, Anne-Marie van Dam

**Affiliations:** 1 Department of Anatomy and Neurosciences, Amsterdam Neuroscience, VU University Medical Center, Amsterdam, The Netherlands; 2 Covalab, Villeurbanne, France; INSERM, FRANCE

## Abstract

The neurodegenerative disease multiple sclerosis (MS) is pathologically characterized by the massive influx of immune cells into the central nervous system. This contributes to demyelination and axonal damage which causes symptoms such as motor and cognitive dysfunctions. The migration of leukocytes from the blood vessel is orchestrated by a multitude of factors whose determination is essential in reducing cellular influx in MS patients and the experimental autoimmune encephalomyelitis (EAE) animal model. The here studied enzyme tissue Transglutaminase (TG2) is present intracellularly, on the cell surface and extracellularly. There it contributes to cellular adhesion and migration via its transamidation activity and possibly by facilitating cellular interaction with the extracellular matrix. Previous data from our group showed reduced motor symptoms and cellular infiltration after using a pharmacological TG2 transamidation activity inhibitor in a rat EAE model. However, it remained elusive if the cross-linking activity of the enzyme resulted in the observed effects. To follow-up, we now characterized two new small molecule TG2 activity inhibitors, BJJF078 and ERW1041E. Both compounds are potent inhibitor of recombinant human and mouse Transglutaminase enzyme activity, mainly TG2 and the close related enzyme TG1. In addition they did not affect the binding of TG2 to the extracellular matrix substrate fibronectin, a process via which TG2 promotes cellular adhesion and migration. We found, that ERW1041E but not BJJF078 resulted in reduced EAE disease motor-symptoms while neither caused apparent changes in pathology (cellular influx), Transglutaminase activity or expression of inflammation related markers in the spinal cord, compared to vehicle treated controls. Although we cannot exclude issues on bioavailability and *in vivo* efficacy of the used compounds, we hypothesize that extracellular TG1/TG2 activity is of greater importance than (intra-)cellular activity in mouse EAE pathology.

## Introduction

Multiple sclerosis (MS) is an inflammatory and demyelinating disease of the central nervous system (CNS) that displays a wide range of clinical symptoms, including sensory, motor and cognitive disabilities [1–3]. In most patients, MS is pathologically characterized by a massive influx of leukocytes into the CNS, which is also observed in the experimental autoimmune encephalomyelitis (EAE) animal model for MS [4]. The infiltrating leukocytes produce inflammatory mediators in the CNS that e.g. activate resident glial cells, induce oligodendrocyte cell death and cause axonal damage [5]. The underlying process of cell infiltration requires the collaboration of numerous factors whose presence is increased during inflammation and that are found within the cells and on their surface [6, 7]. Preventing the interaction of leukocytes with the brain blood vessel endothelium and thus inhibiting the influx of cells into the CNS is the target of recent MS therapies [8, 9].

A novel factor of interest involved in the migration of cells is the enzyme tissue Transglutaminase (TG2) [10]. TG2 is a calcium-dependent protein crosslinking enzyme whose expression and activity can be increased by inflammatory mediators, due to the presence of inflammatory responsive elements in its promotor region [11]. Of interest is that TG2 contributes to cell adhesion and migration via its fibronectin binding domain [10]. In this manner it can act as a co-receptor for β_1_- and β_3_-integrins, which are crucial for the adhesion of cells [12]. The interaction of TG2 and integrins on the cell surface enhances the binding affinity for the extracellular matrix protein fibronectin and promotes cell migration, including that of monocytes [10, 12]. A recent study from our group demonstrated TG2 immunoreactivity in infiltrating leukocytes in MS active lesions. Moreover, we observed that TG2 is present in infiltrating monocytes and contributes to pathology by promoting monocyte migration into the CNS in an experimental MS rat model [13]. In addition, the cross-linking activity of TG2 and possibly other Transglutaminases may contribute to such processes by affecting extracellular matrix deposition and modifications as well as rearrangement of the cytoskeleton [13–15].

Thus, TG2 seems an interesting target in MS, by selectively modulating its enzymatic and/or non-enzymatic activities. The enzymatic transamidating activity of TG2 can be inhibited with chemical compounds to elucidate the contribution of TG2 activity in particular, but also other Transglutaminases, to various cellular processes *in vitro* and *in vivo* and study their link with disease processes.

Due to TG2’s role in cellular adhesion and migration and together with its increased presence during inflammation, we hypothesize that TG2 activity contributes to the clinical outcome of experimental MS. Recent studies from our lab and other groups have shown that TG2 knock-out mice and likewise rats treated with TG2 enzymatic activity inhibiting compounds, showed reduced clinical disease symptoms during EAE [13, 16]. This could, at least partially, be explained by diminished monocyte migration into the CNS [13]. Still, it remains unclear if just TG2 transamidating activity inhibition or also other properties of the used TG2 inhibitor (KCC009), i.e. disturbed fibronectin assembly and therefore the interaction of cells with the ECM, resulted in the observed effects [17]. Therefore, the present study is a follow-up to determine whether the administration of two different TG2 inhibitors affect the clinical outcome of the most commonly used EAE mouse model. For this purpose we chose two different pharmacological compounds; BJJF078 and ERW1041E to inhibit TG2 transamidating activity. The dihydroisoxazol derivative ERW1041E was chosen for to its success to inhibit TG2 activity in a mouse celiac disease and mouse hypoxia experimental model *in vivo*, even though it was known that this inhibitor also inhibits TG1 with a similar potency [18, 19]. The new aminopiperidine derivative BJJF078 has not been extensively characterized yet, but showed very promising TG2 inhibitory capacity over other commonly used TG2 inhibitors in a test tube setting. In the present study, we first characterized the inhibitory capacity of the two TG2 activity inhibitors using various recombinant Transglutaminase proteins. In addition, we measured their ability to inhibit cellular TG2 activity and determined their effect on TG2-fibronectin binding. Ultimately, we studied the expression of various Transglutaminases during mouse EAE and the effect of BJJF078 and ERW1041E treatment on the clinical and pathological outcome of mouse EAE. Overall, we observed that ERW1041E, but not BJJF078 reduced disease symptoms in mouse EAE. As ERW1041E neither inhibits cellular TG2 nor affects the interaction between TG2 and fibronectin, we conclude that extracellular TG2- and possibly TG1-derived transamidating activity contributes to EAE disease.

## Materials and methods

### Inhibition of recombinant Transglutaminase activity

**Fig 1:**
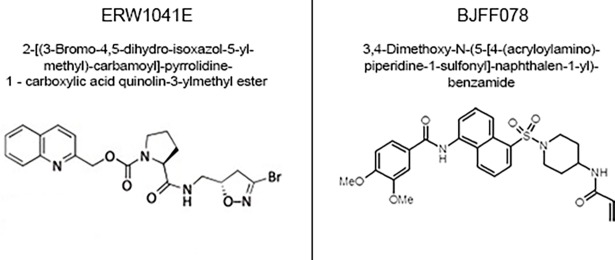
ERW1041E and BJJF078: Name and chemical structure of the TG2 inhibitors.

The potency and specificity of TG2 inhibitors was assessed with various recombinant TGs, as previously described [20, 21]. The reaction buffer (50 mM Tris, 100 mM NaCl, 10 mM CaCl_2_, 5 mM H-Gly-OMe*HCl (glycinemethylestherhydrochloride, Sigma), 5 mM hexadimethinbromide (Sigma), 9.1 mM DTT, pH 7.5) was heated to 37°C in a 96 well plate. Then 50 μM of the TG substrate Abz-NE(CAD-DNP)EQVSPLTLLK-OH (Zedira) was added. 1 μg/ml recombinant TG (human TG1, human (hTG2) / mouse TG2 (mTG2) or human Factor XIII (FXIII); Zedira) were added. The two lyophilized TG2 inhibitors (see Fig 1 for chemical structure) BJJF078 (3,4-Dimethoxy-N-(5-[4-(acryloylamino)piperidine-1-sulfonyl]-naphthalen-1-yl)-benzamide, kindly provided by Dr S. El Alaoui, Covalab, France) and ERW1041E (2-[(3-Bromo-4,5-dihydro-isoxazol-5-ylmethyl)-carbamoyl]-pyrrolidine-1—carboxylic acid quinolin-3-ylmethyl ester, kindly provided by Prof C. Khosla, Stanford University, USA) [18, 22, 23] were dissolved in DMSO (stock solution: 54 mM) and stored at -80°C. These stocks were further diluted and various concentrations of the TG2 inhibitors (0.3 nM to 100 μM), were added to the wells (final DMSO concentration per well: 0.06%). The relative increase in fluorescence emission, correlating with enzymatic activity, was measured over 4 h with a fluorimeter (Excitation: 320nm, emission: 405 nm; Fluostar, GMB).

### Cellular inhibition of TG activity in THP1-cells

Human monocytic THP-1 cells (TIB-202, ATCC) were transduced with a lentiviral vector (pCDH-hTG2, gift from Prof K. Mehta, University of Texas, USA) to overexpress human TG2, to ensure high protein levels in addition to their constitutive expression that can be inhibited. To obtain a stable TG2 overexpressing cell line, transduced cells (THP-1/pCDH-hTG2 (THP-1/TG2)) were positively selected with puromycin and cultured at 37°C and 5% CO_2_ in air in RPMI-1640 medium (Gibco) supplemented with 10% fetal calf serum (FCS; Gibco), and 50 Units/ml of penicillin and 50 μg/ml streptomycin (Gibco).

THP-1/TG2 cells cultured with 3% FCS were differentiated into macrophages with 250 nM phorbol-12-myristate-13-acetate (Sigma) for 48 h hours. Simultaneously, the cells were stimulated with 1 μM retinoic acid (Fluka) as TG2 expression inducer to ensure maximal TG2 expression and activity. The *in vitro* TG activity inhibition assay was performed as previously described [24]. In detail, the cells were washed with serum free medium and incubated for 4 h (37°C, 5% CO_2_) with 1 mM of the TG substrate 5-(biotinamido) pentylamine (BAP, Pierce) in serum free medium. A range of concentrations (0.03 to 100 μM) of the TG2 inhibitors BJJF078 and ERW1041E dissolved in DMSO (final DMSO concentration per well: 0.06%). were added into the wells for 15 min (37°C, 5% CO_2_). As a positive control for this assay similar concentrations of the well characterized TG2 inhibitor Z006 (Zedira) were added to separate wells. Subsequently, 8 μM of the calcium ionophor A23187 was added for 1 h to allow cellular calcium influx which activates (intra-)cellular TG. After washing with cold PBS (supplemented with 1.2 mM calcium and 0.5 mM magnesium), the cells were harvested in ice-cold homogenizing-buffer (50 mM Tris, 150 mM NaCl, 1 mM EDTA, pH 7.5) supplemented with protease inhibitors (100 μM phenylmethylsulfonyl fluoride 1 μg/ml pepstatin, 10 μg/ml aprotinin, 10 μg/ml leupeptin, all Sigma). The total protein concentration was determined with the BCA method (Pierce, according to protocol) and adjusted to the same concentration in all samples. Subsequently, 96-well Maxisorp plates were filled with coating buffer (50 mM Tris, 150 mM NaCl, 5 mM EGTA, 5 mM EDTA, pH 7.4) and 1 μg of protein sample added and incubated on a shaker (overnight, 4°C). After equilibration to room temperature, 200 μl of incubation-buffer (50 mM Tris, 80 mM NaCl, 2.5% bovine serum albumin, 0.01% SDS, 0.01% Tween20, pH7.4) was added to each well. The plate was incubated for 5 minutes on a shaker at room temperature, followed by 2 hours on a shaker at 37°C. After washing with wash-buffer (50 mM Tris, 80mM NaCl, 0.5% bovine serum albumin, 0.01% Tween20, pH 7.4), 100 μl streptavidin conjugated horseradish peroxidase (HRP) (1/5000 in wash-buffer) was added per well and incubated for 1 hour at room temperature on a shaker. This step was followed by a washing step and incubation with the HRP-substrate o-phenylenediamine dihydrochloride (OPD, 0.6 mg/ml in substrate buffer with 38 uM citric acid, 88 uM Na_2_HPO_4_, 4.9 mM H_2_O_2_, pH 5.0). Adding 100 μl OPD-solution starts the colour reaction, which is developed within 30 min and then stopped by adding 100 μl/well 1 M H_2_SO_4_. The optical density at 492 nm was measured.

### Inhibition of TG2 interaction with fibronectin

To determine the potential effect of BJJF078 and ERW1041E on the interaction of TG2 with fibronectin, ELISA experiments were performed as described previously [25]. Briefly, 96 well ELISA plates (Nunc, Maxisorp) were coated with 100 μg/ml mouse anti his-tag monoclonal antibody (Roche) in PBS at 4°C overnight. This was followed by simultaneous washing and blocking with 0.5% BSA in PBS (3x 10 min). Per well 50 μl of a 10 nM human recombinant TG2 (his-tagged, Zedira) solution in PBS was added. Into each well (triplicates) either DMSO (control, 1% final concentration) or TG2 inhibitors (BJJF078, ERW1041E, final concentration 5 and 15 μM in 1% DMSO) were added. As a positive control similar concentrations of TG53, a small molecule that inhibits the interaction between TG2 and fibronectin was added (kindly provided by Prof D. Matei, Northwestern University, Chicago, USA) [25]. To determine possible consequences of the active and inactive enzymatic state of TG2, experiments were performed calcium-free and after addition of 1 mM CaCl_2._ After 1 h incubation at RT, 50 μl of 10 nM FN42-biotin solution (kindly provided by Prof D. Matei) was added to the wells. After an additional 1 h incubation at RT and washing steps (3x 10 min) with 0.5% BSA in PBS, 100 μl streptavidin-HRPO conjugate (from kit opr001, Covalab) was added and incubated for 1 h at RT. This was followed by another series of washes and the colour reaction started with 100 μl of 3,3′,5,5′ tetramethylbenzidine substrate (TMB, from kit opr001, Covalab). After sufficient colour development (15 min), the reaction was stopped with 1M HCl and absorbance was measured at 450 nm.

### Induction of EAE and treatment with TG2 inhibitors

All animal experiments were approved by the animal ethical committee of the VU University Medical Center and carried out within the animal facility of the Vrije Universiteit Amsterdam (protocol number ANW14-02). C57BL/6 (C57BL/6JOlaHsd) mice were purchased from Harlan (The Netherlands) at an age of 10 weeks and acclimatized to the new environment with water and food access ad libitum for 2 weeks before EAE induction. Humane endpoints were taken into account to prevent unnecessary suffering of the animals.

Active EAE was induced with the myelin oligodendrocyte glycoprotein peptide amino acids 35-55 (MOG_35-55_) from a EAE induction kit (Hooke laboratories), according to the provided protocol. In short, mice were injected subcutaneously in the upper and lower back with 100 μl of an emulsion of MOG_35-55_ in complete Freund’s adjuvant (CFA, containing heat inactivated Mycobacterium tuberculosis) each. At 3 hours and 26 hours after the subcutaneous injection of the emulsion, mice received an intraperitoneal injection of 150 ng pertussis toxin dissolved in PBS (volume: 0.1 ml). Disease symptoms and bodyweight were assessed daily starting from day 7 after immunization according to the following scale: 0 no disease, 0.5: partial tail weakness, 1: tail paralysis, 2: hind limb weakness, 3: partial hind limb paralysis, 4: complete hind limb paralysis, 5: moribund, dead [26].

The mice were injected twice daily with BJJF078 (n = 11) or ERW1041 (n = 12), starting from the onset of EAE symptoms until the end of the experiment (day 21/day 28 after immunization). The EAE animals were injected intraperitoneally with an inhibitor dose of 25 mg/kg bodyweight (i.e. ~100 μl) containing 20% DMSO, while the corresponding control groups (n = 12 and n = 10) were injected with PBS in 20% DMSO.

### Tissue processing

Animals were sacrificed between day 21 and day 28 post EAE induction. Mice were anaesthetized by intraperitoneal injection of ketamine and xylazine and sacrificed by decapitation. Liver, spleen and spinal cord tissue were carefully dissected and snap frozen in liquid nitrogen and stored at -80 C until use. Coronal cryo-sections (10 μm) were cut from the cervical, thoracic and lumbar spinal cord tissue (embedded in OCT tissue tek, VWR Chemicals), dehydrated and stored at -80°C until use.

### *In situ* TG activity in tissue sections

*In situ* TG activity detection in post-mortem tissue from EAE mice was performed as described previously [27]. Thawed unfixed cryo-sections of the cervical, thoracic and lumbar spinal cord were incubated in buffer (100 mM Tris-HCl, pH 7.4, 5 mM CaCl_2_, 1 mM DTT, pH 7.4) for 20 min at room temperature. The incubation was continued for 30 min at 37°C in buffer supplemented with 50 μM of the TG substrate BAP (Pierce). Some sections had 100 μM of the TG2 inhibitor Z006 (Zedira) added to the buffer in both incubation steps to determine TG2 specificity of the derived signal in the assay. Sections were subsequently washed with MilliQ water, air-dried and fixed for 10 min with acetone. After washes in TBS, endogenous peroxidase activity was blocked for 15 min with 0.3% hydrogen peroxide and 0.1% sodium azide in TBS. Subsequently, sections were blocked with 3% bovine serum albumin in TBS containing 0.5% TritonX-100 (TBS-T). This step was followed by incubation with HRP-labelled avidin-biotin complex (ABC complex 1:400; Vector Laboratories) for 1 h at RT and detection with the chromogen 3,3-diaminobenzidine (DAB, Sigma) and nuclear counterstaining with haematoxylin, if applicable. Sections were dehydrated in graded ethanol solutions and cleared in xylene before coverslipping with Entellan mounting medium (Merck Millipore).

### Immunohistochemistry

Immunohistochemical staining was performed on cervical, thoracic and lumbar spinal cord sections from EAE mice treated with vehicle, BJJF078 or ERW1041E. The sections were brought back to room temperature and fixed with acetone for 10 min.

#### Single immunohistochemical staining

For single immunohistochemical stainings (CD3, CD45, CD68), endogenous peroxidase activity was blocked for 15 min with 0.3% hydrogen peroxide and 0.1% sodium azide in TBS. Sections were subsequently blocked with 3% bovine serum albumin in TBS containing 0.5% TritonX-100 (TBS-T). The sections were incubated with primary antibodies (Table 1) at 4°C overnight. After subsequent 2 h incubation with appropriate biotinylated IgGs (1/400, Jackson), the procedure was continued with a 1 h incubation with HRP-labelled avidin-biotin complex (ABC complex 1:400, Vector ABC elite kit, Vector laboratories) and the protocol was followed as described for the *in situ* TG activity staining above. Semi-quantitative analysis of cellular infiltrates per cell type was performed by unbiased manual evaluation of stained lesion areas per spinal cord section.

**Table 1:** Primary antibodies used for immunohistochemistry.

Target	Host	Dilution	Supplier	Code/Clone
CD3	Rat	1/200 (DAB) 1/100 (FL)	Serotec	MCA500G
CD45	Rat	1/10	Dept. MCBI, VUmc, Amsterdam, Netherlands [28] (gift)	MP33
CD68	Rat	1/500 (DAB) 1/1000 (FL)	Serotec	MCA1957
TG1	Goat	1/100	Santa Cruz	sc-18129
TG2	Mouse	1/100	Prof T. J. Johnson, Sheffield University, UK (gift)	Clone IA-12

FL: fluorescent; DAB: 3,3-diaminobenzidine

#### Bright field immunohistochemical double labelling

Co-labelling of TG1 and CD45 was performed for bright field microscopy. After the blocking of endogenous peroxidase activity blocked with 0.3% hydrogen peroxide and 0.1% sodium azide in TBS for 15 min, the sections were subsequently blocked with 3% bovine serum albumin in TBS-T. The sections were incubated with both primary antibodies (Table 1) at 4°C overnight. After 30 min incubation with ImmPRESS alkaline phosphatase coupled anti goat IgGs (Vector laboratories), a 2 h incubation with biotinylated mouse anti rat IgGs followed (1/400, Jackson). Subsequently, the sections were incubated for 1 h incubation with HRP-labelled avidin-biotin complex (ABC complex 1:400, Vector ABC elite kit, Vector laboratories). Ultimately, colour reaction was performed first with liquid permanent red (LPR, Dako) and then with DAB. Images acquired with a spectral imaging camera (Nuance) are shown pseudo coloured in the style of fluorescent staining to increase visual contrast.

#### Fluorescent immunohistochemical double labelling

Immunohistochemical fluorescent co-labelling of the immune cell markers CD3, CD45 and CD68 with TG2 (Table 1) required pre-treatment with a mouse on mouse blocking kit (M.O.M.; Vector Laboratories) according to the manufacturer’s guidelines. Briefly, the sections were blocked for 1 h with blocking reagent. This was followed with incubation with primary antibody (TG2 and either CD3, CD45 or CD68) at 4°C overnight. After 2 h incubation with AF594 or AF488 labelled appropriate IgGs (1/400, Molecular Probes), the sections were coverslipped with Vectashield hard containing DAPI (Vector laboratories).

### RNA isolation and semi-quantitative RT-PCR

Total RNA from snap frozen spinal cord (pooled cervical, thoracic and lumbar region) was isolated using Trizol (Invitrogen) according to the manufacturer’s protocol. RNA concentration and purity was determined with a NanoDrop 2000 spectrophotometer (Thermo Fisher Scientific). Purity was accepted with a 260/230 nm absorbance ratio of 2.00–2.20 and a 260/280 ratio of 1.90.-2.00. For reverse transcription into cDNA, 1 μg of RNA was used with the High-Capacity cDNA Reverse Transcription kit (Life Technologies), using 50 pmol oligo-dT16 primers, according to the manufacturer’s instructions. For quantitative real-time PCR (qPCR), Power SYBR Green Master Mix (Applied Biosystems) was used according to manufacturer’s protocol. Intron-spanning primers (Table 2) were designed with Primer-BLAST [29], purchased from Eurogentec and used at 3.75 pmol per 12.5 ng cDNA in each reaction. Subsequently, qPCR was performed according to the previously published MIQE guidelines [30]. The qPCR reaction was performed in a total volume of 20 μl in MicroAmp Optical 96-well Reaction Plates on a StepOnePlus Real-Time PCR system (Applied Biosystems). The thermal cycling conditions were an initial 10 min at 95°C followed by 50 cycles of 15 s at 95°C and 1 min at 60°C. The reaction specificity was verified by a melt curve analysis. The relative expression level of the target genes was determined by the LinRegPCR software (http://www.hfrc.nl, downloads, applications, lin reg PCR, version 2017) using the following calculation N0 = Nq/ECq (N0 = target quantity, Nq = fluorescence threshold value, E = mean PCR efficiency per amplicon, Cq = threshold cycle [31]. The obtained values were normalized to glyceraldehyde-3-phosphate-dehydrogenase expression (GAPDH) [32, 33].

**Table 2:** Oligonucleotide primers used for cDNA amplification.

Protein / gene	Reference sequence, NCBI	Sequences forward (fwd) and reverse (rev)	Amplicon length (base pairs)
FXIII / F13A1	NM_028784.3	fwd: 5’-CAGCAATGGTGAATGCCAAGGA-3’ rev: 5’-GCTGATGGAGGGATGCCGTA-3’	85
GAPDH	NM_008084.2	fwd: 5’-GGAGAAACCTGCCAAGTATG-3’ rev: 5’-TCCTCAGTGTAGCCCAAGATG-3’	90
IFNγ / IFNG	NM_008337.4	fwd: 5’-GTTTGAGGTCAACAACCCAC-3’ rev: 5’-ATCAGCAGCGACTCCTTTTC-3’	116
IL-1β / IL1b	NM_008361.4	fwd: 5’-GCCACCTTTTGACAGTGATG-3’ rev: 5’- CTTCTCCACAGCCACAATGA-3’	184
IL-1ra / IL1RA	M57525.1	fwd: 5’-TGACAGTGGAACGGAATGAC-3’ rev: 5’-GTATCCCAGATTCTGAAGGCT-3’	98
iNOS / NOS2	NM_010927.3	fwd: 5’-ACATCGACCCGTCCACAGTAT-3’ rev: 5’-CAGAGGGGTAGGCTTGTCTC-3’	177
TG1 / Tgm1	NM_001161715.1	fwd: 5’-ACCAGCAGTGGCATCTTC-3’ rev: 5’-ATGAAAGGTGTGTCATACTTC-3’	88
TG2 / Tgm2	NM_009373.3	fwd: 5’-GGGCCTTCTCATCGAACCAG-3’ rev: 5’-CAGGACCCGGATCTTGATTTC-3’	91
TG3 / Tgm3	NM_009374.3	fwd: 5’- ACATCAGCACCAAGGCAGTAGG -3’ rev: 5’- CCTCGAAGATGTTGCGCCGAAA-3’	152
TG6 / Tgm6	NM_001289747.1	fwd: 5’- CAGCAGTGGTAGGAGTGACAG -3’ rev: 5’- CTCTTGGAAGGGGTTATGTTG-3’	187
TNFα / TNFa	NM_013693.2	fwd: 5’-GTAGCCCACGTCGTAGCAAAC-3’ rev: 5’-GGCACCACTAGTTGGTTGTCTT-3’	116

### Statistical analysis

Normal distribution of the data was determined with the Shapiro-Wilk test (GraphPad Prism, Version 6). If normally distributed, subsequent student T-test or, if not normally distributed, Mann-Whitney U test or generalized linear model analysis was applied (GraphPad Prism or IBM SPSS Statistics, Version 22). Significant differences were considered to be present if p-values were below 0.05.

## Results

### BJJF078 and ERW1041E inhibited recombinant Transglutaminase activity

**Fig 2:**
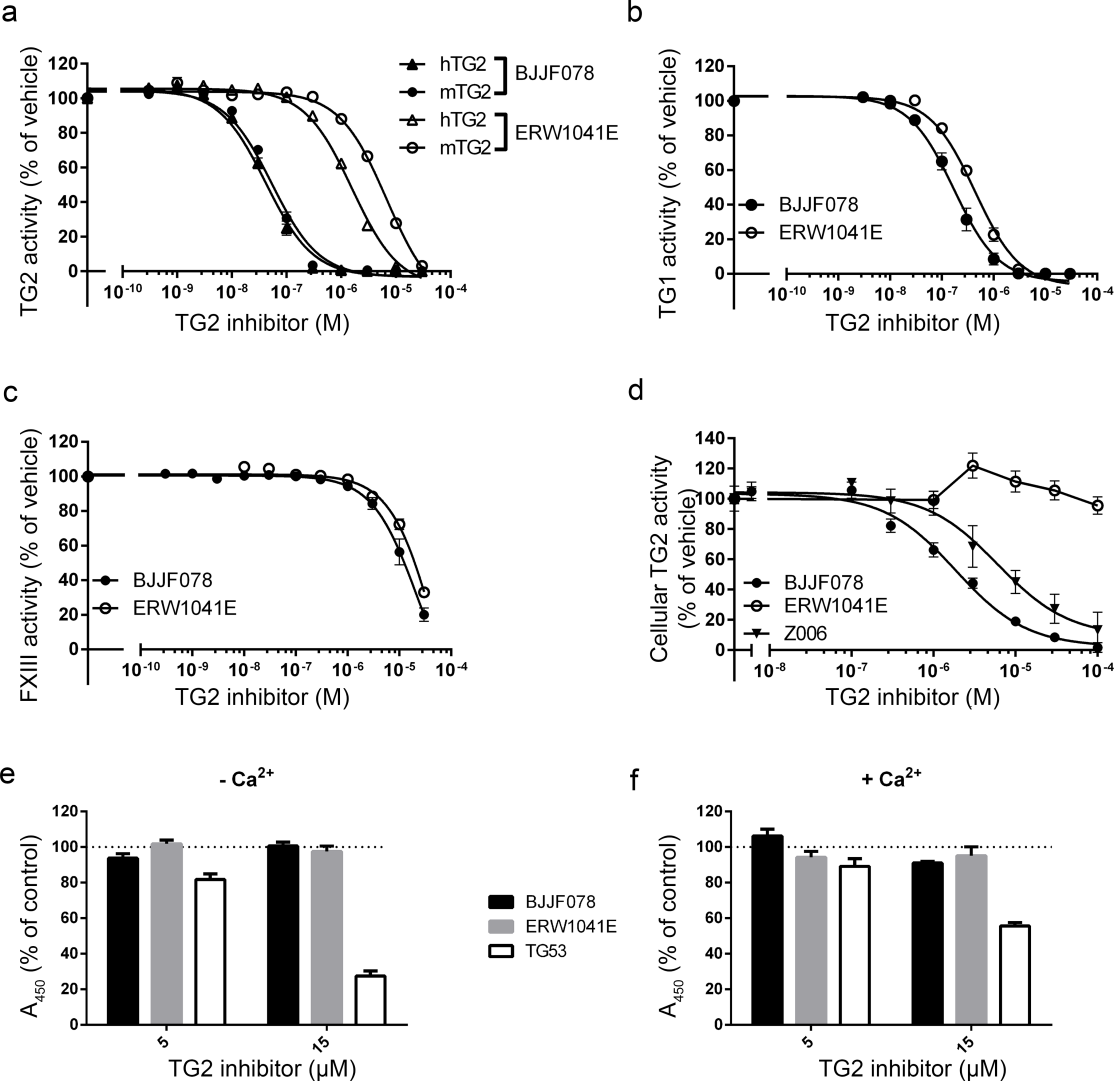
Inhibition of TG activity and TG2-fibronectin interaction by BJJF078 and ERW1041E in solution and *in vitro*. (a) BJFF078 and ERW1041E dose-dependently inhibited human (hTG2) and mouse (mTG2) recombinant TG2 activity. BJJF078 and ERW1041E inhibited also (b) human recombinant TG1 and (c) human recombinant FXIII. (d) Cellular activity of human TG2 in THP1 cells was dose-dependently inhibited by BJJF078 and Z006 but not by ERW1041E. (e,f) Effect of BJJF078 and ERW1041E on TG2 binding to fibronectin without (e) or with added calcium (f). n = 1–3 independent experiments with triplicates each.

Test tube measurements were performed to determine the inhibitory potency and specificity of BJJF078 and ERW1041E towards TG1, TG2, and FXIII enzymatic activity. Although the homology of human and mouse TG enzymes is high (84%, protein BLAST, NCBI), these TG2 inhibitors were specifically designed to inhibit human TG2 activity. As the present study intended to use them in a mouse model, we compared the inhibitory effect of the compounds on recombinant mouse and human TG2 activity (Fig 2A). IC_50_ values of BJJF078 were 41 and 54 nM, for human and mouse TG2, respectively. ERW1041E proved less potent and required μM concentrations to inhibit TG2 activity. Furthermore it was slightly more potent to inhibit human than mouse TG2 activity (IC_50_ of 1.6 μM vs 6.8 μM, respectively). Other members of the TG family tested included human TG1 and Factor XIII. The IC_50_ of BJJF078 to inhibit TG1 was 0.16 μM and it was 0.44 μM for ERW1041E (Fig 2B). Furthermore, the IC_50_ values of BJJF078 and ERW1041E to inhibit FXIII activity were 22 μM and 52 μM, respectively (Fig 2C).

### BJJF078 and not ERW1041E inhibited cellular TG2 activity *in vitro*

We determined if BJJF078 and ERW1041E were able to inhibit cellular TG activity. TG2 overexpressing THP-1 macrophages were stimulated to ensure high amounts of active enzyme that can be pharmacologically inhibited. BJJF078 effectively reduced TG2 transamidating activity in this setup, with an IC_50_ of 1.8 μM (Fig 2D, closed circles). ERW1041E instead proved to have no effect on TG2 activity in this experimental setup (Fig 2D, open circles). The well-established TG2 inhibitor Z006 was used as positive control (Fig 2D, triangles) and showed an IC_50_ of 6 μM, which is in a similar range as BJJF078.

### BJJF078 and ERW1041E did not interfere with TG2 binding to fibronectin

To measure effects of the TG2 activity inhibitors on the interaction between TG2 and fibronectin (fragment FN42), an ELISA experiment was conducted. Neither BJJF078 nor ERW1041E interfered with binding of TG2 to FN42, whereas TG53, a small molecule inhibitor designed to interrupt TG2-fibronectin binding resulted in a dose-dependent reduction of TG2-fibronectin binding irrespective of the presence of calcium (Fig 2E and 2F).

### ERW1041E modestly reduced EAE disease symptoms

**Fig 3:**
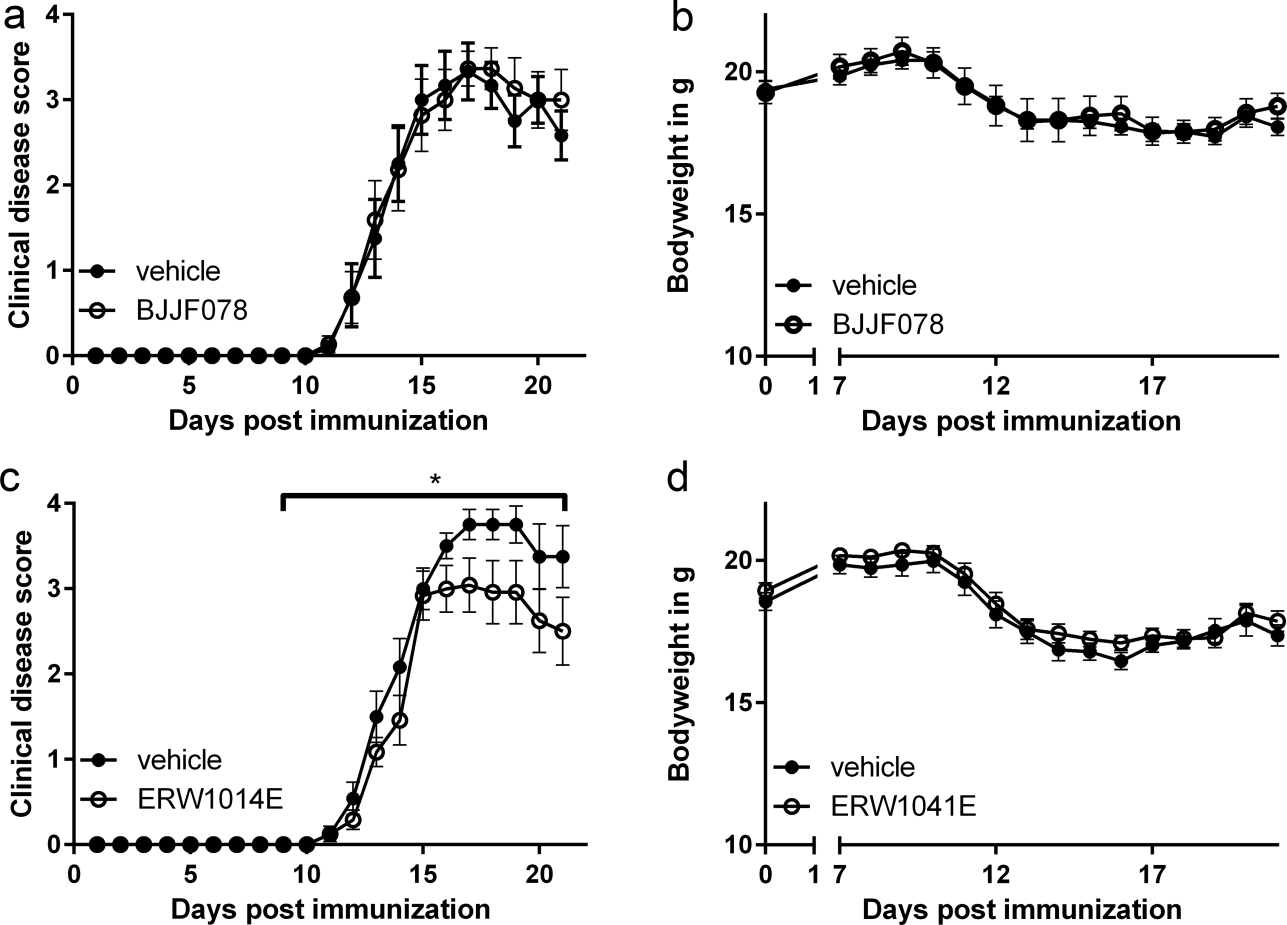
Disease scores and bodyweight of EAE animals treated with BJJF078 or ERW1041E. (a) BJJF078 did not affect motor symptoms or (b) bodyweight of EAE affected mice compared to vehicle treated animals. (c) ERW1041E reduced motor symptoms significantly without (d) affecting the loss of bodyweight of EAE affected mice. n = 11–12 animals per treatment group. Statistics: generalized linear model, *P<0.05.

We determined the effect of BJJF078 and ERW1041E on the outcome of EAE in C57BL/6 mice *in vivo* when treated from the first signs of disease motor symptoms onward. BJJF078-treated mice showed no differences in the severity of motor symptoms compared to the vehicle-treated control group (Fig 3A). Furthermore, no differences in the loss of body weight was observed (Fig 3B). In contrast, treatment with ERW1041E resulted in a modest but significant reduction in the severity of motor symptoms compared to vehicle treated mice (Fig 3C). During disease development the progress of motor symptoms is alike, but the average maximal disease score reached in the ERW1041E (score 3.4) treated animals was lower than in the vehicle control group (score 3.9). The loss of bodyweight was similar in both groups and thus not affected by ERW1041E treatment (Fig 3D). No obvious adverse effects of the compound injections were observed *in vivo* or by post-mortem analysis of the abdomen.

### TGs mRNA and protein in the spinal cord of EAE mice

**Fig 4:**
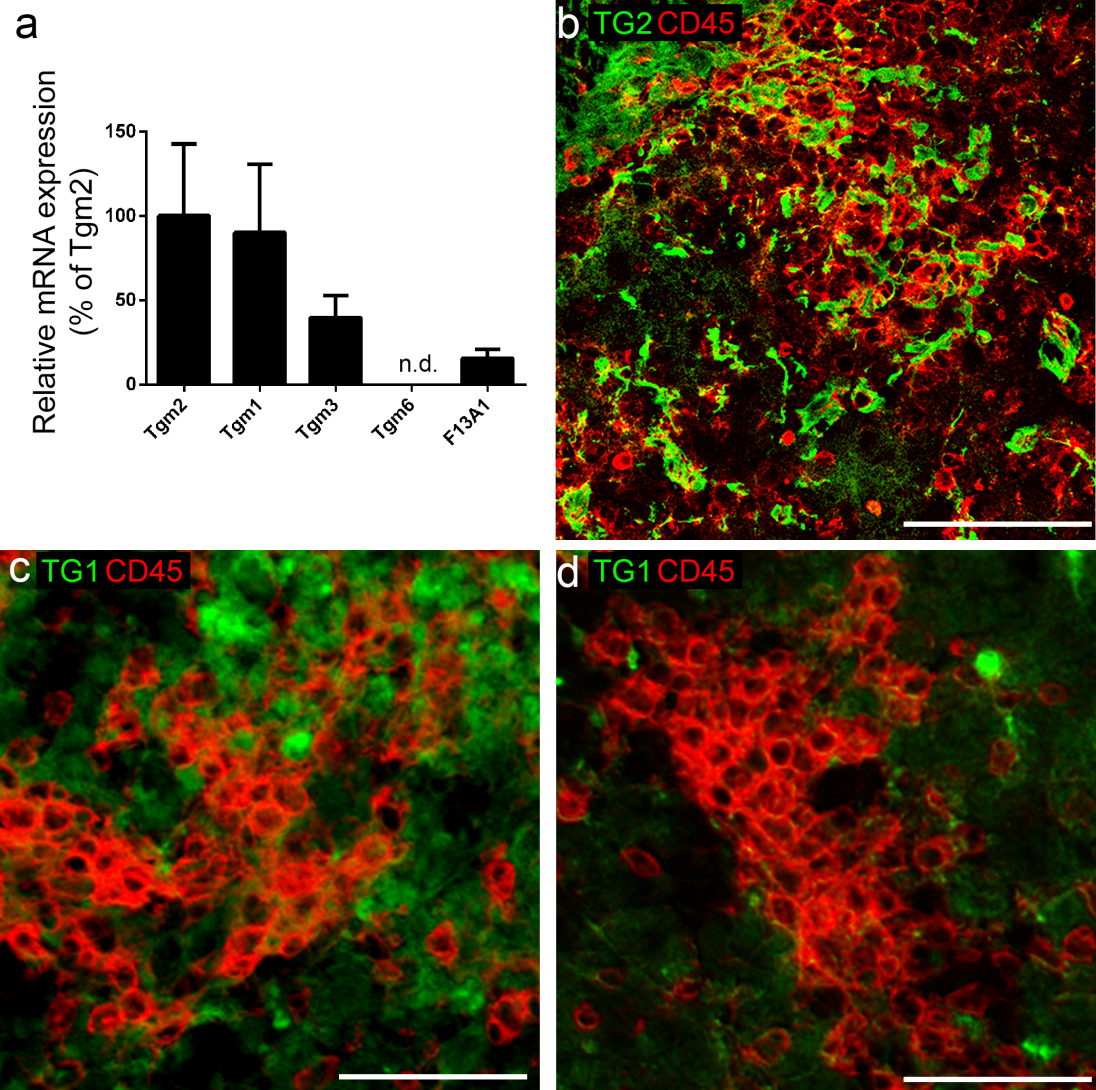
Transglutaminases mRNA, and TG1 and TG2 immunoreactivity in post-mortem spinal cord tissue of EAE animals. (a) EAE spinal cord mRNA levels for Tgm1, Tgm2, Tgm3, Tgm6 (n.d. = not detectable) and F13A1, (b) TG2 immunoreactivity (green) associates with CD45 (red) stained EAE lesions whereas TG1 immunoreactivity (green) is found in some lesions (d) but not in others (d). Scale bar: 25 μm. n = 9 animals/group.

The mRNA levels of various TG family members were measured in the spinal cord of vehicle-treated EAE animals as their presence could, in addition to TG2, contribute to EAE pathology. Tgm1 and Tgm2 were equally expressed, while Tgm3 and F13A1 showed lower mRNA levels and Tgm6 expression was below the detection limit (Fig 4A). Additionally, we identified TG1 and TG2 protein in the spinal cord using immunohistochemical staining. At first, the infiltrated leukocytes in the spinal cord lesions were visualized with the general leukocyte marker CD45, indicating EAE lesion formation. TG2 immunoreactivity was present in all inflammatory lesions of the spinal cord and closely associated with CD45^+^ cells (Fig 4B). Whereas TG1 immunoreactivity was less associated with CD45^+^ cells (Fig 4C and 4D). Furthermore, about half of the spinal cord lesions show the presence of TG1 immunoreactivity (Fig 4C) while the other lesions are almost devoid of TG1 (Fig 4D).

### TG activity in spinal cord sections of EAE mice was not affected by ERW1041E or BJJF078

**Fig 5:**
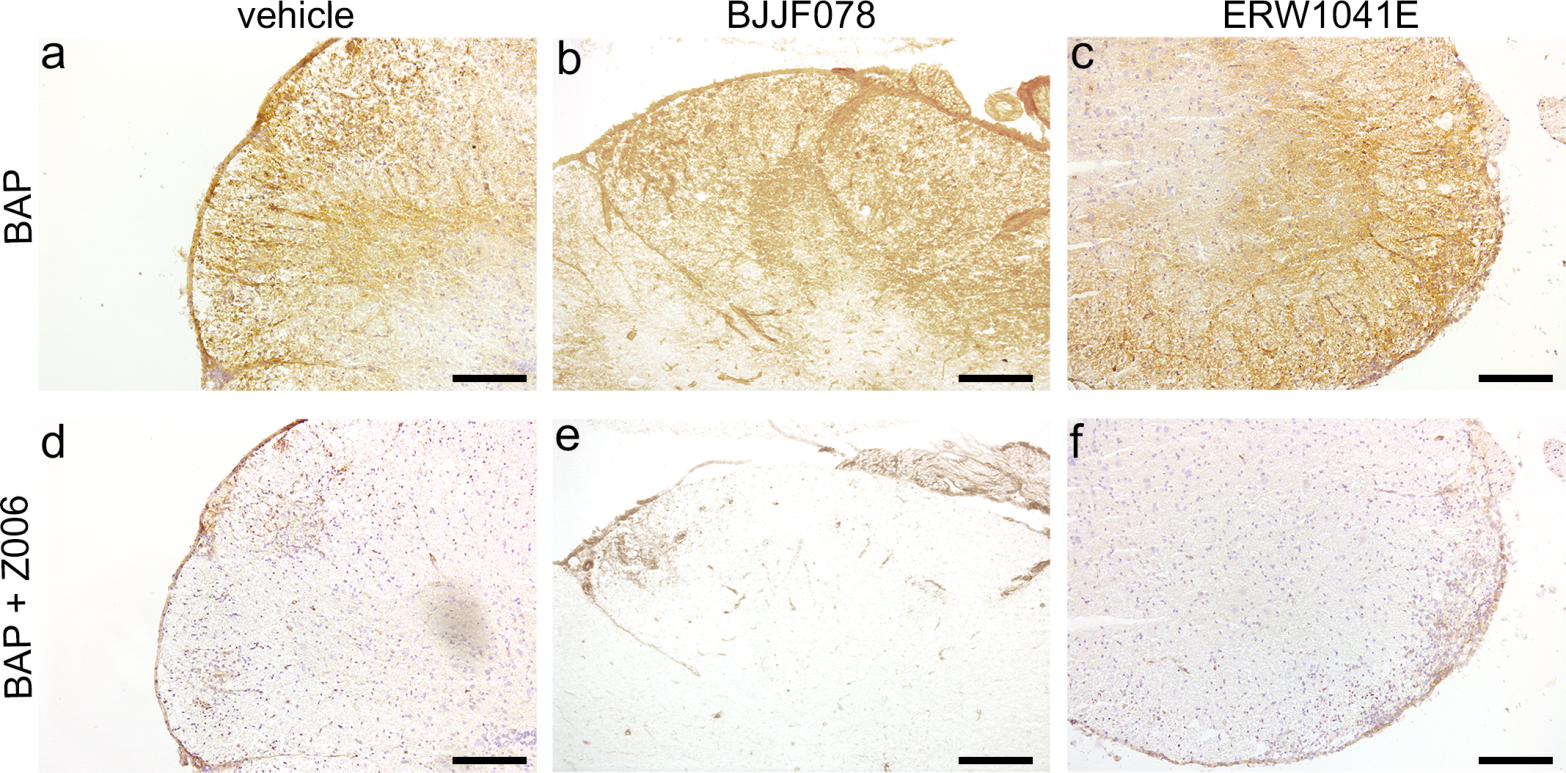
TG activity in spinal cord post-mortem tissue derived from EAE animals treated with vehicle, BJJF078 or ERW1041E. (a-f) *In situ* TG activity was detected in spinal cord sections derived from (a,d) vehicle, (b,e) BJJF078 and (c,f) ERW1041E treated mice. (d-f) Co-incubation of the sections with the TG2 inhibitor Z006 diminished TG activity. Scale bar: 50 μm. n = 11 or 12 animals/group.

In addition to the presence of TGs, also their activity was analysed. *In situ* incorporation of the general TG substrate BAP in spinal cord sections of EAE mice allowed us to determine the level of TG enzymatic activity. To take a possible short *in vivo* half-life of BJJF078 into account which is unknown, the animals received the last injection 30–60 min before they were sacrificed. Some inter-individual variation of TG activity signal was found in the animals, which correlated to the amount of lesions per section. However, the TG substrate BAP was readily incorporated in similar amounts in spinal cord sections from vehicle, BJJF078 and ERW1041E treated mice, independent of treatment (Fig 5A–5C). This TG related signal, that is associated with the inflammatory lesions, was clearly reduced when the highly specific TG2 inhibitor Z006 was co-applied to the sections, indicating the source of the signal to be mainly TG2 (Fig 5D–5F).

### Inflammatory cell marker and TG2 expression in the spinal cord

**Fig 6:**
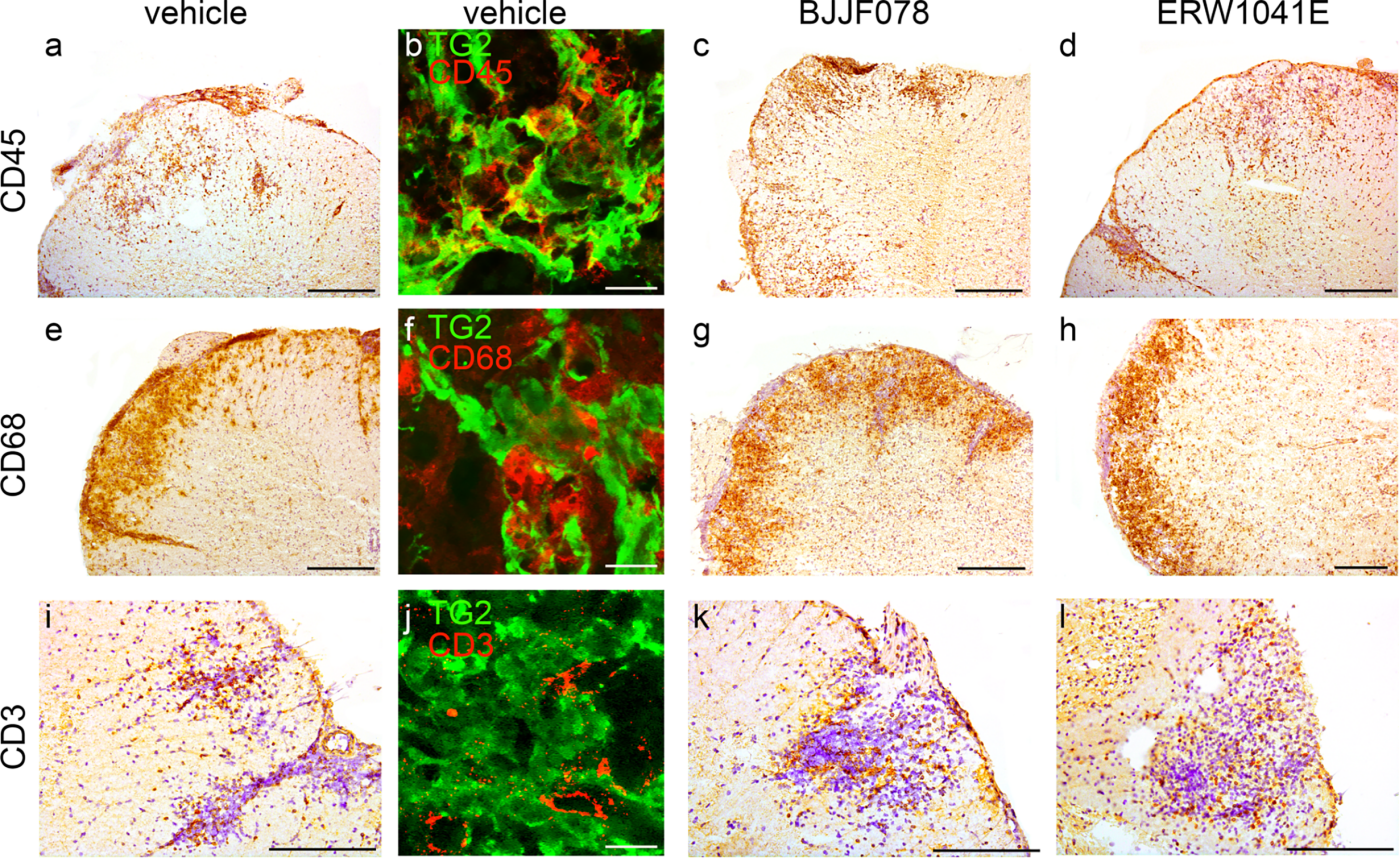
Cell infiltrates and TG2 expression in the spinal cord of BJJF078 or ERW1041E treated EAE mice. (a-d) BJJF078 and ERW1041E did not affect the infiltration of CD45 positive leukocytes into the spinal cord. Also (e-h), CD68 positive phagocytic cells and (i-l) CD3 positive T cells were observed in similar amount in vehicle and TG2 inhibitor treated animals. (b, f, j) show co-labelling of TG2 (green) with the immune cells makers (red) (b) CD45, (f) CD68 and (j) CD3. Scale bar: 100 μm, except for b,f,j: 50 μm. n = 9–12 animals/group.

Inflammatory lesions in the spinal cord of EAE mice were identified by detection of CD45 positive leukocytes (Fig 6A–6D), CD68 positive macrophages (Fig 6E–6H) and CD3 positive T cells (Fig 6I–6L). The relative amount of these inflammatory cells was not affected by treatment with BJJF078 or ERW1041E upon visual inspection (Fig 6C, 6D, 6G, 6H, 6K and 6L) or after semi-quantification (Table 3). Co-labelling of the CD markers (red) with TG2 (green) revealed that CD45 presence (Fig 6B) was closely associated with that of TG2. The immunoreactivity of CD68 (Fig 6F) was associated with TG2 to a lesser extent whereas CD3 immunoreactivity seemed to be found in patches with no TG2 expression (Fig 6J).

**Table 3:** Semi-quantitative analysis of CD45, CD68 and CD3 expression in the spinal cord of EAE mice treated with vehicle or TG2 activity inhibitors.

	CD45 range (average)	CD68 range (average)	CD3 range (average)
**vehicle (control)**	+ to +++(++)	- to +++(++)	- to +(-/+)
**BJJF078**	+ to +++(++)	- to +++(++)	- to +(-/+)
**ERW1041E**	+ to +++(++)	- to +++(++)	- to +(-/+)

-: no positive cells, + few positive cells, ++: intermediate amounts of positive cells, +++: high amounts of cells/ cellular clusters, in 3 sections per animal (cervical, lumbar and thoracic spinal cord) of 9–12 animals/group

### Expression of inflammatory mediators in spinal cord of ERW1041E-treated EAE mice

**Fig 7:**
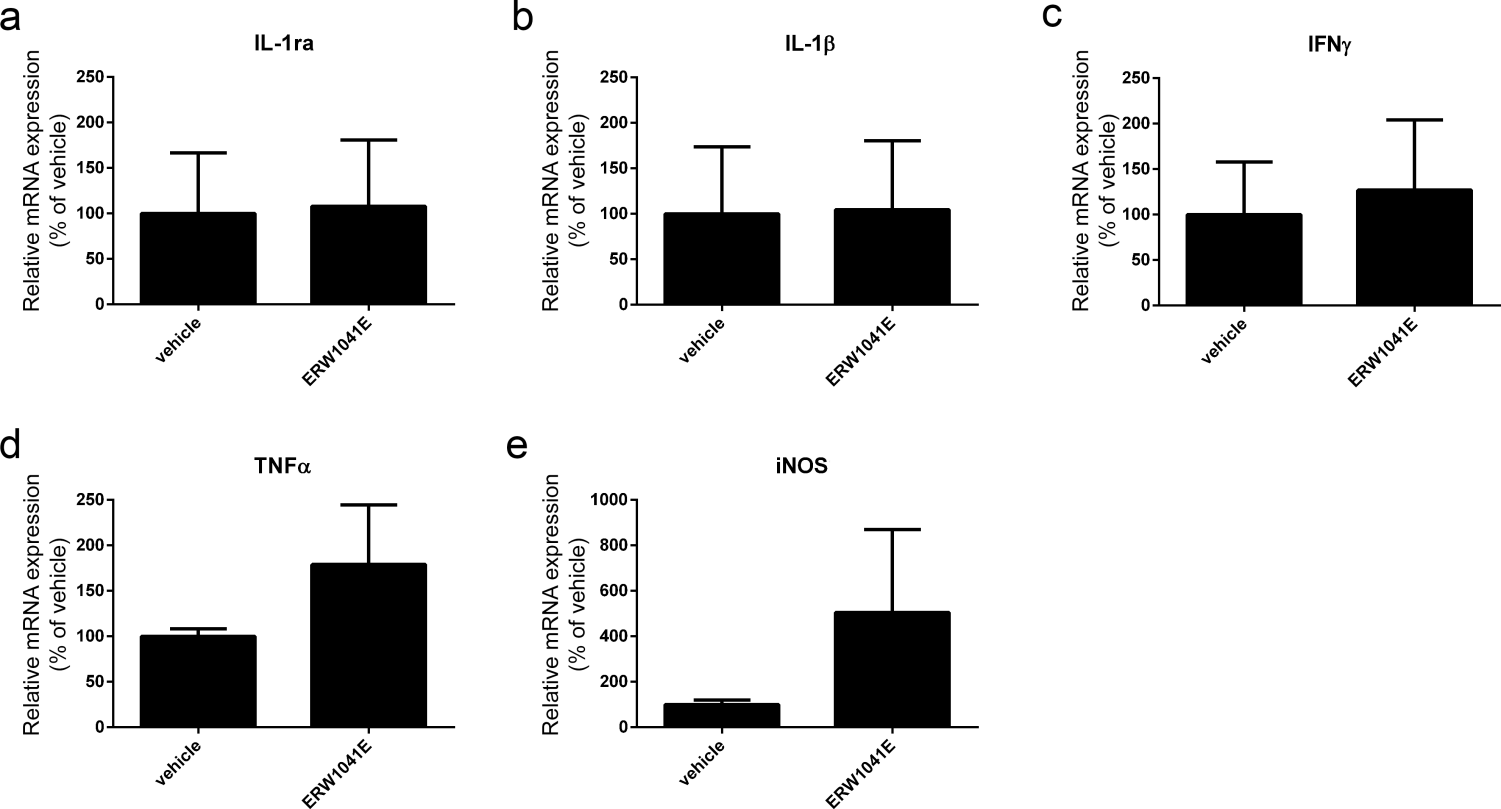
qPCR analysis of gene expression associated with inflammation in the spinal cord of ERW1041E and vehicle only treated EAE mice. mRNA levels of inflammatory markers (a) IL-1ra, (b) IL-1β, (c) IFNγ, (d) TNFα and (e) iNOS were not significantly changed in spinal cord of ERW1041E animals compared to the vehicle treated group. n = 9–12 animals/group. Statistical analysis: student T-test or, if not normally distributed: Mann-Whitney U test.

ERW1041E treated EAE mice showed less motor symptoms than vehicle-treated mice, which was not corroborated by an altered leukocyte pathology in the spinal cord as shown by immunohistochemistry. We therefore analysed the mRNA levels of inflammation-related genes which could be affected by TG2 inhibition in this model. RNA levels of the following factors were measured: IL-1ra, IL-1β, IFNγ, TNFα and iNOS. Whereas most mRNA levels were not affected by ERW1041E treatment, TNFα and iNOS mRNA levels were slightly but not significantly enhanced after ERW1041E treatment (Fig 7A–7E).

## Discussion

In the present study we used two irreversible TG2 activity inhibitors to measure their effect on disease symptoms, severity and pathology in a mouse EAE model for multiple sclerosis. Our previous studies identified TG2 as a possible therapeutical target in a rat chronic relapsing EAE model in which TG2 inhibition reduced cellular infiltration as well as disease symptoms [13, 34]. Moreover, EAE induced in C57BL/6 mice has previously been shown to be, at least partly, mediated by TG2 [13]. The present work extends these observations of TG2 inhibition using new pharmacological compounds in mouse EAE, to determine whether Transglutaminase activity is of relevance in the commonly used mouse MS model in the C57BL/6 strain. The treatment of the animals with the compound ERW1041E led to a significant reduction in motor symptom severity compared to vehicle treated animals, while this was not observed after treatment with the compound BJJF078.

Although many TG2 inhibitors have been presented over the years, their use in animal models is scarce. The dihydroisoxazol derivate ERW1041E was chosen in this study for its previous proven efficacy *in vivo* [18, 19] and the new aminopiperidine derivative BJJF078 showed very promising TG2 inhibitory ability with recombinant proteins (data not published) and the inhibition of cellular TG2 activity. The characterization of both compounds using recombinant human and mouse TG2 revealed a similar inhibitory capacity of recombinant TG2 and is reflecting the 84% protein sequence homology of TG2 in both species (protein BLAST, NCBI).

Despite the previous *in vivo* results with ERW1041E that are presumably TG2 mediated [19], this inhibitor is not specific for TG2 alone but shows equipotency for TG1, as we showed to be the case for BJJF078 as well, while another TG member of the TG family, i.e. FXIII, is inhibited to a lower degree. However, it has to be pointed out that the affinities of the tested recombinant Transglutaminase enzymes for the substrate used in the assay vary and consequently it is not possible to make a direct comparison of the IC_50_ values between the Transglutaminase isotypes.

Subsequently, we studied the expression of various Transglutaminases in murine EAE spinal cord and found equal levels of Tgm1 and Tgm2 and lower levels of Tgm3, Tgm6 and F13A1 mRNA. Although we could hardly detect Tgm6 mRNA, other recent data showed TG6 protein expression in human MS and mouse EAE spinal cord lesions [35], which could indicate a low turn-over of the protein. By studying the *in situ* Transglutaminase activity in the spinal cord sections of EAE mice, we observed that most of the measured TG activity is derived from TG2. This is supported by the observation that the spinal cord lesions are consistently associated with TG2 but not necessarily with TG1 expression. Furthermore, we observed that TG2 is more associated with infiltrated CD45^+^ leukocytes than TG1 in the lesions. Concludingly, it is likely that the reduction in mouse EAE symptoms after treatment with ERW1041E is due to inhibition of TG2 activity. Nevertheless,we cannot fully exclude that off-target effects and/or inhibition of TG1 and other Transglutaminases activity is involved as well.

So far very little is known about the mode of action of TG2 and possibly other TGs during EAE. It remained unknown if extracellularly and/or (intra-)cellularly located TGs contribute to EAE disease and which function(s) of the enzymes are of importance. We observed that BJJF078, in contrast to ERW1041E [36], can inhibit cellular TG2 activity although in EAE mice no measurable effect was found. These observations suggest that extracellular TG2 activity seemed to be more relevant than (intra-)cellular TG2 activity to mediate EAE motor symptom development.

Surprisingly, ERW1041E treatment did neither affect the leukocyte influx nor the production of inflammatory mediators in the murine EAE spinal cord. This is in contrast to the previously observed results of pharmacological TG2 activity inhibition with the TG2 inhibitor KCC009 in rat EAE, although both inhibitors are structurally related and belong to the dihydroisoxazol type [13]. Of interest in this respect is that KCC009 can interfere with fibronectin assembly [13, 17], whereas the interaction of TG2 and fibronectin was not affected by either BJJF078 or ERW1041E. This may at least partly explain why no change in leukocyte infiltration was observed in the present study. Another explanation of the different outcome of ERW1041E and the previously used KCC009 treatment, is the use of a mouse EAE model instead of the rat chronic relapsing EAE model. The pathogenesis of this mouse model in C57BL/6 mice is more depended on lymphocytes than monocytes [37, 38], the suspected main source for TG2 [13]. Moreover, the non-cellular site of action of ERW1041E might suggest a different mechanism than purely cellular TG and especially TG2 activity inhibition aiding cell migration. A possible mechanism of action could lie in TG activity affecting extracellular matrix deposition and stiffness [14, 39]. This process might be altered by the inhibition of TGs *in vivo*, irrespective of the effect on cell migration or production of inflammatory mediators [15]. This could also explain why BJJF078 and thus cellular TG inhibition did not exert any effect in this model.

An additional explanation for the lack of effect of BJJF078 could be its limited bioavailability. A previous successful *in vivo* dose and treatment scheme of ERW1041E was used [18], but no *in vivo* bioavailability information on BJJF078 is available yet. Hence, it might be possible that BJJF078 is (1) not distributed to the site of interest or (2) has much shorter half-life compared to the proposed 12 h inhibitory *in vivo* half-life of ERW1041E. Therefore we cannot exclude that BJJF078 might be useful as an *in vivo* TG activity inhibitor once its pharmacokinetic properties are revealed and optimized. We can thus not eliminate that cellular TG1 and TG2, in addition to extracellular TG enzymes, may contribute to EAE.

To summarize, our data indicate that BJJF078 and ERW1041E are both potent inhibitors of human and mouse TG2 and TG1 activity but do not interfere with TG2-fibronectin binding. Interestingly, in mouse EAE, ERW1041E and not BJJF078 treatment is able to reduce motor symptoms. Although we cannot exclude issues on bioavailability and *in vivo* efficacy of the inhibitors used, we hypothesize that surface/extracellular TG1/TG2 activity is of greater importance than (intra-)cellular TG1/TG2 activity to mediate mouse EAE pathogenesis. Furthermore, this effect is independent of TG2-fibronectin binding which is neither affected by ERW1041E nor by BJJF078, but the potential side and mechanism of action of ERW1041E remain elusive. Subsequent studies could focus on delineating the process of surface or extracellular TG1/TG2 transamidating activity in this EAE model, which might be of interest to reduce cellular infiltration in EAE and MS.
