# Correction: Characterization of Transglutaminase 2 activity inhibitors in monocytes *in vitro* and their effect in a mouse model for multiple sclerosis

**DOI:** 10.1371/journal.pone.0209522

**Published:** 2018-12-13

**Authors:** Navina L. Chrobok, John G. J. M. Bol, Cornelis A. Jongenelen, John J. P. Brevé, Said El Alaoui, Javier Fidalgo-Lopez, Guy Fournet, Benoît Joseph, Micha M. M. Wilhelmus, Benjamin Drukarch, Anne-Marie van Dam

Dr. Javier Fidalgo-Lopez, Dr. Guy Fournet, and Dr. Benoît Joseph are not included in the author byline. The contributions of these authors are as follows: Design and synthesis of BJJF078. Please view the correct author byline, affiliations, and citation here:

Navina L. Chrobok^1^, John G. J. M. Bol^1^, Cornelis A. Jongenelen^1^, John J. P. Brevé^1^, Said El Alaoui^2^, Javier Fidalgo-Lopez^2, 3☯^, Guy Fournet^3☯^, Benoît Joseph^3☯^, Micha M. M. Wilhelmus^1^, Benjamin Drukarch^1^, Anne-Marie van Dam^1^

☯ These authors contributed equally to this work.

1 Department of Anatomy and Neurosciences, Amsterdam Neuroscience, VU University Medical Center, Amsterdam, The Netherlands, 2 Covalab, Villeurbanne, France, 3 Institut de Chimie et Biochimie Moléculaires et Supramoléculaires, Université Claude Bernard—Lyon 1, Villeurbanne, France

Chrobok NL, Bol JGJM, Jongenelen CA, Brevé JJP, El Alaoui S, Fidalgo-Lopez J, et al. (2018) Characterization of Transglutaminase 2 activity inhibitors in monocytes *in vitro* and their effect in a mouse model for multiple sclerosis. PLoS ONE 13(4): e0196433. https://doi.org/10.1371/journal.pone.0196433

There is an error in the fifth sentence of the Materials and methods section under the subheading “Inhibition of recombinant Transglutaminase activity”. The correct sentence is: The two lyophilized TG2 inhibitors (see Fig 1 for chemical structure) BJJF078 (3,4-Dimethoxy-N-(5-[4-(acryloylamino)piperidine-1-sulfonyl]-naphthalen-1-yl)-benzamide, see S1 Appendix, synthetized and provided by Covalab, Villeurbanne, France and Institut de Chimie et Biochimie Moléculaires et Supramoléculaires, Villeurbanne, France) and ERW1041E (2-[(3-Bromo-4,5-dihydro-isoxazol-5-ylmethyl)-carbamoyl]-pyrrolidine-1—carboxylic acid quinolin-3-ylmethyl ester, kindly provided by Prof C. Khosla, Stanford University, USA) [18, 22, 23] were dissolved in DMSO (stock solution: 54 mM) and stored at -80°C.

**Fig 1:**
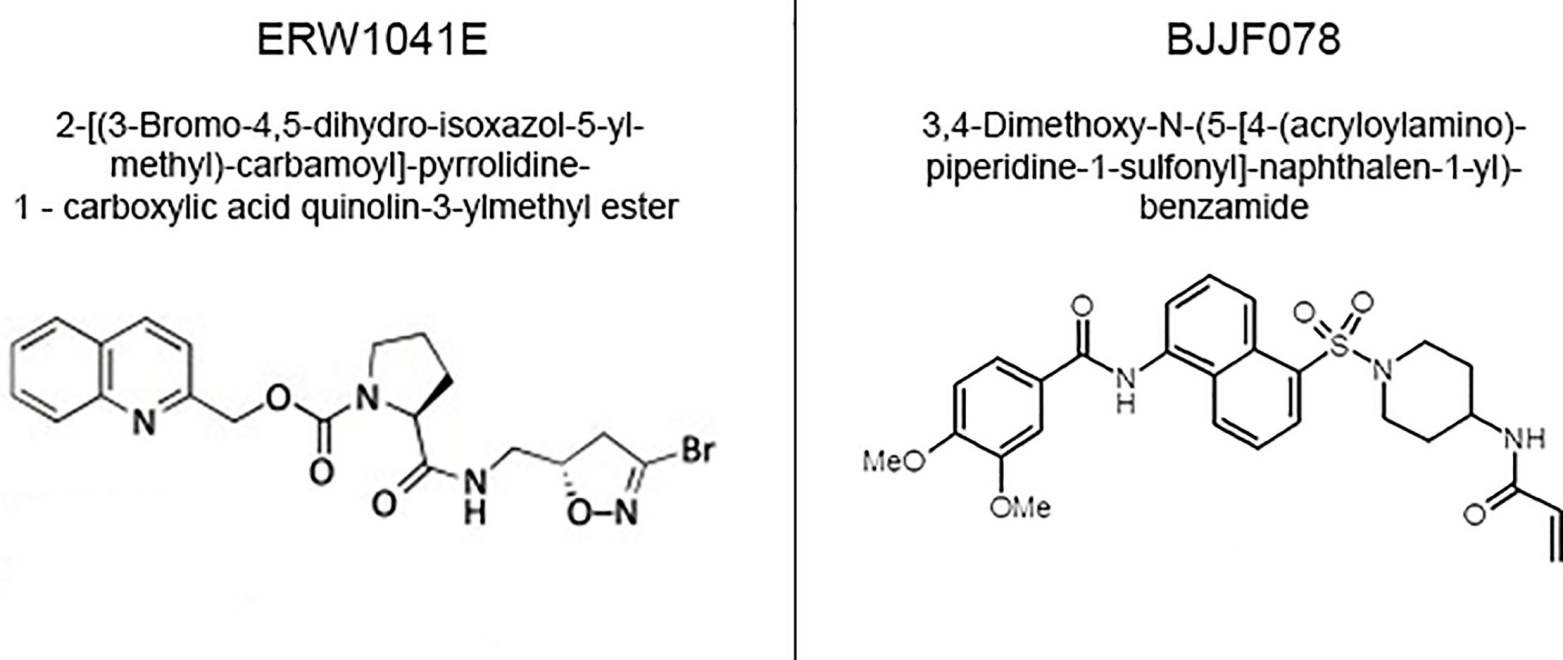
ERW1041E and BJJF078: Name and chemical structure of the TG2 inhibitors.

In Fig 1, the heading BJFF078 should be BJJF078. The authors have provided a corrected version here.

## Supporting information

S1 Appendix Description of the synthesis of compound BJJF078. (DOCX)
