# Supplementary material for: Correction: Characterization of Transglutaminase 2 activity inhibitors in monocytes in vitro and their effect in a mouse model for multiple sclerosis
Source: PLoS One. 2018 Dec 13;13(12):e0209522. doi: 10.1371/journal.pone.0209522 (PMC6292628; doi:10.1371/journal.pone.0209522)
Supplement: S1 Appendix — (DOCX) [file pone.0209522.s001.docx]

**S1 Appendix**

**Synthesis of BJJF078**

*Step 1*: to a solution of the commercially available 5-amino-1-naphthalenesulfonic acid (**1**) (CAS: 84-89-9) (1 equivalent) in pyridine (0.67 M) was added 3,4-dimethoxybenzoyl chloride (1.2 equivalents) and the resultant solution was stirred at 100ºC for 20 h. The solvent was removed under vacuum and the crude material was taken up in methanol (twice) to obtain a precipitate. The latter was filtered to give 5-[(3,4-dimethoxybenzoyl)amino]naphthalene-1-sulfonate pyridinium salt (**2**) as a solid. Yield: 56%; mp: > 210ºC (washing MeOH); ^1^H NMR (300 MHz, DMSO-*d_6_*) δ (ppm): 3.85 (s, 3H, CH_3_), 3.86 (s, 3H, CH_3_), 7.11 (d, *J* = 8.5 Hz, 1H, H_Ar_), 7.45 (dd, *J* = 7.2, 8.5 Hz, 1H, H_Ar_), 7.48-7.57 (m, 2H, H_Ar_), 7.68 (d, *J* = 2.0 Hz, 1H, H_Ar_), 7.75 (dd, *J* = 2.0, 8.4 Hz, 1H, H_Ar_), 7.89-8.01 (m, 4H, H_Ar_), 8.41-8.51 (m, 1H, H_Ar_), 8.81 (dd, *J* = 1.5, 7.7 Hz, 1H, H_Ar_), 8.87 (d, *J* = 5.2 Hz, 2H, H_Ar_), 10.30 (s, 1H, NH, NH); ^13^C NMR (75 MHz, DMSO-*d_6_*) δ (ppm): 55.6 (CH_3_), 55.7 (CH_3_), 111.0 (CH), 111.1 (CH), 121.2 (CH), 124.1 (CH), 124.4 (CH), 124.5 (CH), 124.9 (CH), 125.1 (CH), 126.2 (CH), 126.5 (C), 127.1 (2 CH), 129.8 (C), 130.2 (C), 133.9 (C), 142.5 (2 CH), 144.2 (C), 145.9 (CH), 148.4 (C), 151.6 (C), 165.6 (C=O); MS (ESI): m/z = 386.3 [M-PyrH]^-^.

*Step 2*: to a suspension of the pyridinium salt **2** (1 equivalent) in DMF (0.59 M) was added dropwise thionyl chloride (1.5 equivalents). The reaction was stirred at room temperature for 16 h. The reaction mixture was poured into ice-water. The resulting solid was quickly filtered, washed twice by cold water and dried over P_2_O_5_ under vacuum. The freshly moisture sensitive crude 5-[(3,4-dimethoxybenzoyl)amino]naphthalene-1-sulfonyl chloride (**3**) was not further purified and was used directly in the next step. Yield: 87%.

*Step 3*: to a solution of the ammonium salt **4** (1 equivalent) in DMF (0.1 M) was added *N*,*N*-diisopropylethylamine (DIPEA, 3 equivalents) at room temperature, followed by the addition of **3** (1,1 equivalents) at 0ºC. The reaction mixture was stirred at room temperature for 16 h. The mixture was poured dropwise into water and the resulting precipitate was filtered, washed with 1N HCl and washed again with water (twice). The crude solid was dried over P_2_O_5_ under vacuum then purified by column chromatography on silica gel (solvent: MeOH/CH_2_Cl_2_ 5:95) to give 3,4-dimethoxy-*N*-(5-[4-(acryloylamino)piperidin-1-sulfonyl]naphthalen-1-yl)benzamide (**BJJF078**). Yield: 64%; mp: 125-128ºC (washing EtOH); ^1^H NMR (300 MHz, DMSO-*d_6_*) δ (ppm): 1.31-1.48 (m, 2H, CH_2_), 1.76-1.88 (m, 2H, CH_2_), 2.83-2.95 (m, 2H, CH_2_), 3.59-3.79 (m, 3H, CH + CH_2_), 3.87 (s, 6H, 2 x CH_3_), 5.55 (dd, *J* = 2.7, 9.7 Hz, 1H, =CH_2_), 6.04 (dd, *J* = 2.7, 17.1 Hz, 1H, =CH_2_), 6.16 (dd, *J* = 9.7, 17.1 Hz, 1H, -CH=), 7.14 (d, *J* = 8.6 Hz, 1H, H_Ar_), 7.63-7.83 (m, 5H, H_Ar_), 8.06 (d, *J* = 7.6 Hz, 1H, NH), 8.20 (d, *J* = 6.4 Hz, 1H, H_Ar_), 8.32 (d, *J* = 8.3 Hz, 1H, H_Ar_), 8.56 (d, *J* = 8.5 Hz, 1H, H_Ar_), 10.45 (s, 1H, NH); ^13^C NMR (101 MHz, DMSO-*d_6_*) δ (ppm): 30.9 (2 x CH_2_), 44.0 (2 x CH_2_), 44.8 (CH), 55.6 (CH_3_), 55.7 (CH_3_), 111.0 (CH), 111.1 (CH), 121.3 (CH), 122.9 (CH), 124.6 (CH), 125.2 (CH=), 125.3 (=CH_2_), 126.2 (C), 127.8 (CH), 128.8 (C), 129.9 (CH), 130.0 (CH), 130.5 (C), 131.7 (CH), 133.5 (C), 135.1 (C), 148.4 (C), 151.8 (C), 163.8 (C=O), 165.7 (C=O); MS (ESI): m/z = 524.2 [M+H]^+^; HRMS (ESI): calcd for C_27_H_30_N_3_O_6_S [M+H]^+^, 524.1850; found, 524.1835.

Schematic representation of BJJF078 synthesis
